# Establishment and Characterization of a New Intrahepatic Cholangiocarcinoma Cell Line Resistant to Gemcitabine

**DOI:** 10.3390/cancers11040519

**Published:** 2019-04-11

**Authors:** Chiara Varamo, Caterina Peraldo-Neia, Paola Ostano, Marco Basiricò, Chiara Raggi, Paola Bernabei, Tiziana Venesio, Enrico Berrino, Massimo Aglietta, Francesco Leone, Giuliana Cavalloni

**Affiliations:** 1 Department of Oncology, University of Turin, 10100 Torino, Italy; chiara.varamo@unito.it (C.V.); massimo.aglietta@unito.it (M.A.); francesco.leone@unito.it (F.L.); 2 Laboratory of Tumor Inflammation and Angiogenesis, Department of Oncology, Center for Cancer Biology, KU Leuven, B3000 Leuven, Belgium; 3 Cancer Genomics Lab, Fondazione Edo ed Elvo Tempia, 13900 Biella, Italy; caterina.peraldoneia@ircc.it (C.P.-N.); paola.ostano@gmail.com (P.O.); 4 Division of Medical Oncology, Candiolo Cancer Institute, FPO-IRCCS, 10060 Candiolo, Torino, Italy; marco.basirico@ircc.it; 5 Center for Autoimmune Liver Diseases, Humanitas Clinical and Research Center, 20089 Rozzano, Italy; chiara.raggi@unifi.it; 6 Dept. Medicina Sperimentale e Clinica, Università di Firenze, 50100 Florence, Italy; 7 Flow Cytometry Center, Candiolo Cancer Institute FPO-IRCCS, 10060 Candiolo, Torino, Italy; paola.bernabei@ircc.it; 8 Molecular Pathology Lab, Unit of Pathology, Candiolo Cancer Institute FPO-IRCCS, 10060 Candiolo, Torino, Italy; tiziana.venesio@ircc.it (T.V.); enrico.berrino@ircc.it (E.B.); 9 Department of Medical Sciences, University of Turin, Corso Dogliotti 14, 10126 Turin, Italy

**Keywords:** cholangiocarcinoma, gemcitabine, drug resistance, preclinical models, gene expression profiling

## Abstract

Intrahepatic cholangiocarcinoma (ICC) is one of the most lethal liver cancers. Late diagnosis and chemotherapy resistance contribute to the scarce outfit and poor survival. Resistance mechanisms are still poorly understood. Here, we established a Gemcitabine (GEM) resistant model, the MT-CHC01R1.5 cell line, obtained by a GEM gradual exposure (up to 1.5 µM) of the sensitive counterpart, MT-CHC01. GEM resistance was irreversible, even at high doses. The in vitro and in vivo growth was slower than MT-CHC01, and no differences were highlighted in terms of migration and invasion. Drug prediction analysis suggested that Paclitaxel and Doxycycline might overcome GEM resistance. Indeed, in vitro MT-CHC01R1.5 growth was reduced by Paclitaxel and Doxycycline. Importantly, Doxycycline pretreatment at very low doses restored GEM sensitivity. To assess molecular mechanisms underlying the acquisition of GEM resistance, a detailed analysis of the transcriptome in MT-CHC01R1.5 cells versus the corresponding parental counterpart was performed. Transcriptomic analysis showed that most up-regulated genes were involved in cell cycle regulation and in the DNA related process, while most down-regulated genes were involved in the response to stimuli, xenobiotic metabolism, and angiogenesis. Furthermore, additional panels of drug resistance and epithelial to mesenchymal transition genes (*n* = 168) were tested by qRT-PCR and the expression of 20 genes was affected. Next, based on a comparison between qRT-PCR and microarray data, a list of up-regulated genes in MT-CHC01R1.5 was selected and further confirmed in a primary cell culture obtained from an ICC patient resistant to GEM. In conclusion, we characterized a new GEM resistance ICC model that could be exploited either to study alternative mechanisms of resistance or to explore new therapies.

## 1. Introduction

Cholangiocarcinoma (CCA) is a malignant tumor that originates from the epithelial cells of the biliary tract. It is classified as extrahepatic (ECC) and intrahepatic (ICC) cholangiocarcinoma depending on the anatomical onset of the tumor [1]. Due to its aggressiveness, CCA is characterized by a high mortality rate and poor prognosis. It is a relatively rare cancer whose diagnosis is difficult; most CCA patients are diagnosed at a state of unresectable disease and their overall 5-year survival is about 5%. The therapeutic options are limited and the only curative treatment is surgical resection with a 5-year survival reported around 20% to 50% [2]. In unresectable locally advanced and metastatic disease, no significant improvement in treatment has been achieved over the last decade. Cisplatin and Gemcitabine (GEM) combination is considered the standard of treatment, according to a phase III trial [3]. Other chemotherapy combinations have been investigated (Oxaliplatin, Capecitabine or Fluoropyrimidine, Epirubicin, Taxanes, Mytomicin), but mainly in phase II of retrospective trials, with small and heterogeneous cohorts of patients. Unfortunately, the expected overall survival at 12 months from diagnosis is about 50% [4,5]. The phenomenon of resistance to the current therapies is inevitable; indeed, CCA is able to escape several known tolerance mechanisms [6]. These mechanisms include the acquisition of genetic mutations, changes in the metabolism responsible for drug inhibition and degradation, activation of survival signaling pathways, and alterations of drug uptake and export [7]. Further, the tumor microenvironment plays an important role in chemoresistance by releasing specific factors, altering the sensitivity to chemotherapy [8]. 

The development of chemoresistance, in particular to GEM, represents the major point of failure in CCA treatment, responsible for progression and mortality of patients. 

GEM is a deoxycytidine nucleoside analog, which is introduced in the cells by nucleoside transporters and then phosphorylated by deoxicitydine kinase (dCK), and acts by directly interfering with DNA synthesis, ribonucleotide reductase (RRMs) inhibition, and blocking cell cycle progression at the G1/S-phase transition [9]. GEM resistance has been well studied in pancreatic cancer. In particular, the involvement of several factors has been demonstrated, such as deficiencies in drug uptake, activation of alternative pathways involved in DNA repair, resistance to apoptosis, or in epithelial-mesenchymal transition (EMT) [10]. However, few information concerning GEM resistance is available in CCA. Therefore, an extensive study of the mechanisms underlying CCA GEM resistance may help to identify key molecules involved in the onset of this phenomenon. 

In the present study, we established a GEM resistant CCA cell line, the MT-CHC01R1.5 and provide a biological and molecular characterization. Our data offer a useful model of chemoresistance either to study new mechanisms of resistance or to explore alternative therapeutic strategies in CCA. 

## 2. Results

### 2.1. Biological Characterization of a New Established Gemcitabine Resistant Cholangiocarcinoma Cell Line

First, the GEM resistant clone, named MT-CHC01R1.5, was obtained by exposing parental cells intermittently to escalating doses of the drug, starting from 10 nM of GEM to 1.5 μM for 9 months. After 3 months of being continuously cultured in the presence of 1.5 μM, the MT-CHC01R1.5 cells were considered stable. 

**Figure 1:**
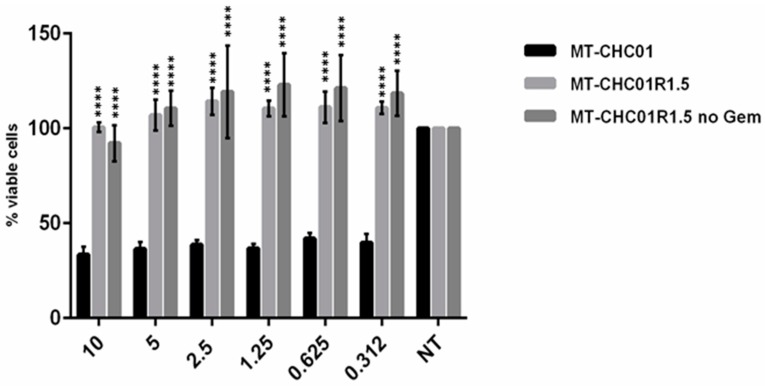
Gemcitabine (GEM) effects on survival: Treatment was performed at 72 h using different GEM doses. MT-CHC01: parental cells; MT-CHC01R1.5: GEM resistant cells; MT-CHC01R1.5 no Gem: MT-CHC01R1.5 resistant clone deprived from GEM for 10 days and re-exposed to escalating doses of the drug for 72 h. All the experiments were conducted in three independent experiments in quadruplicate. Error bars represent mean and SD. **** *p* = 0.00001.

Next, in order to confirm GEM resistance, both MT-CHC01 and MT-CHC01R1.5 cells were treated with escalating doses of the drug. Figure 1 shows the in vitro growth inhibition of sensitive and resistant cells after GEM treatment; MT-CHC01R1.5 cells presented a remarkable resistance at high doses (until 10 μM) compared to the parental cell line. GEM growth inhibition in MT-CHC01 cells was not dose-dependent. Interestingly, MT-CHC01R1.5 resistance was irreversible; indeed, after GEM deprivation for 10 days, the resistant clone was refractory when re-exposed to the drug for 72 h (Figure 1). 

**Figure 2:**
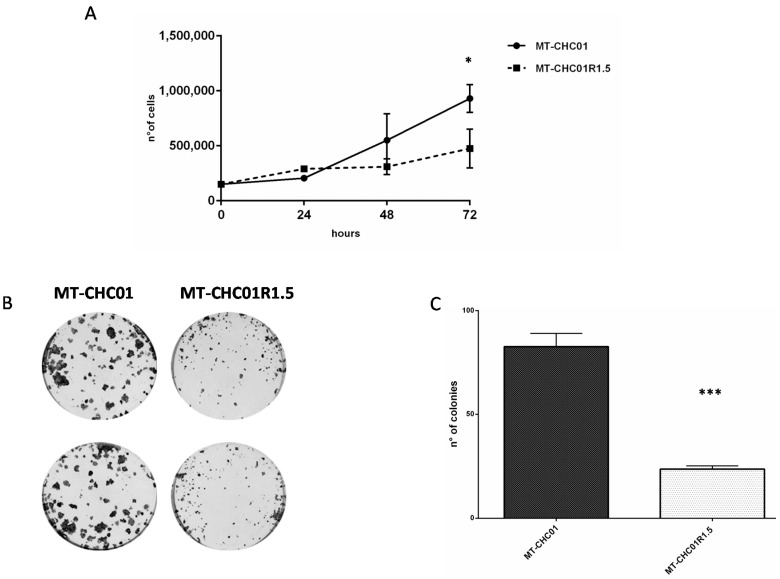
Differences in cell growth and colony formation. (**A**) Growth curves of MT-CHC01 and MT-CHC01R1.5 cells: 1.5 × 10^5^ cells were plated in 6-well plates in triplicate in three different experiments in optimal medium. Viable cells were counted at 24, 48, and 72 h after seeding. (**B**) Colony formation assay on MT-CHC01 and MT-CHC01R1.5 cells: Representative images of colony formation after 10 days of seeding. (**C**) Quantification of colony formation. Colonies formed by more than 10 cells were counted in triplicate in three different experiments. There is a statistically significant difference in terms of the number of colonies between MT-CHC01 and MT-CHC01R1.5 (*p* = 0.0001). Error bars represent the mean and SD. * *p* = 0.01, *** *p* = 0.0001.

To determine the population doubling time of the GEM resistant clone, we compared the cell growth curves of parental MT-CHC01 and resistant MT-CHC01R1.5 cells. As shown in Figure 2A, the doubling time of MT-CHC01 and MT-CHC01R1.5 cells was 25.3 and 49.1 h, respectively. The difference in terms of cell growth was statistically significant at 72 h (*p* = 0.01). We confirmed the number of viable cells by quantifying ATP, a key indicator of metabolically active cells (CellTiter GLO assay). Indeed, the MT-CHC01 proliferation rate was 1.73 ± 0.25 compared to its resistant counterpart (Figure S1).

The difference was highly appreciable in the colony formation assay (Figure 2B); indeed, the number of colonies with more than 10 cells was significantly different between the GEM sensitive and resistant clones (82.66 ± 6.42 for MT-CHC01 vs. 23.66 ± 1.5 for MT-CHC01R1.5; *p* = 0.0001). 

**Figure 3:**
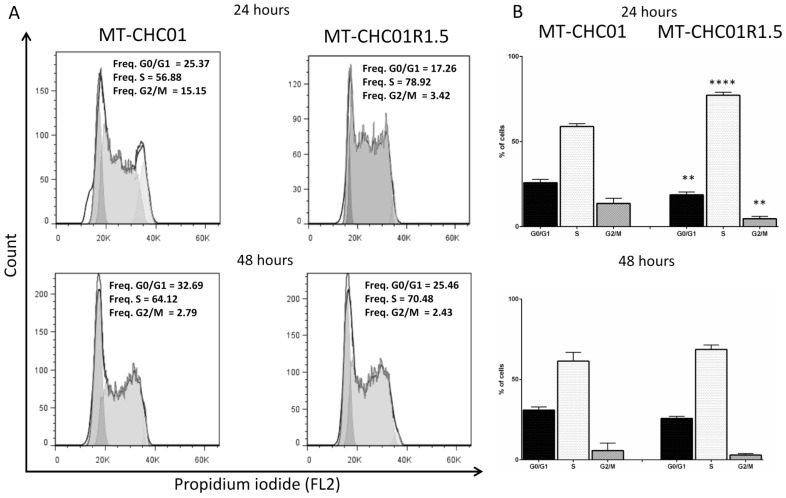
Cell cycle analysis: (**A**) Representative flow cytometry histograms of cell populations in MT-CHC01 and MT-CHC01R1.5 cells assigned to different cell cycle phases (G0/G1, S, and G2) based on the intensity of PI staining (reflecting DNA content), gated on a single cell population. Cells were plated in optimal culture conditions (MT-CHC01R1.5 also in the presence of GEM) for 24 and 48 h and flow cytometry was performed, and data analyzed using FlowJo software. (**B**) Statistical analysis of the cell cycle distribution was conducted for three independent experiments. A significant increase of phase S was found in MT-CHC01R1.5 (*p* = 0.0001), while a significant decrease of phases G0/G1 and G2/M was revealed (*p* = 0.001) after 24 h. No significant differences were found after 48 h. ** *p* = 0.001 and **** *p* = 0.00001.

Cell cycle distribution in parental and GEM resistant cells was determined by using flow cytometric analysis. As shown in Figure 3, a significant S phase expansion was observed in MT-CHC01R1.5 cells compared to MT-CHC01 parental cells after 24 h of culture (77.24% vs. 58.82% of cells, respectively, *p* < 0.001), reflecting the enhanced doubling time of resistant clone. At the same time, a significant decrease of G0/G1 (25.85% vs. 18.70%, *p* = 0.001) and G2/M (13.63% vs. 4.62%, *p* = 0.001) phases was observed in resistant cells. No differences were found after 48 h of culture.

From the morphological point of view, no appreciable difference was observed between parental and resistant cells. In order to verify whether GEM resistance conferred a more invasive phenotype, migration and invasion tests were carried out. MT-CHC01 and MT-CHC01R1.5 cells were added in an invasion chamber under serum free conditions in the absence or in the presence of extracellular matrix. After 24 h of incubation, no differences in both the number of migrated or invaded cells was observed, thus suggesting that in our model, the chemotherapy resistance was not linked to a more aggressive phenotype in terms of mobility potential.

**Figure 4:**
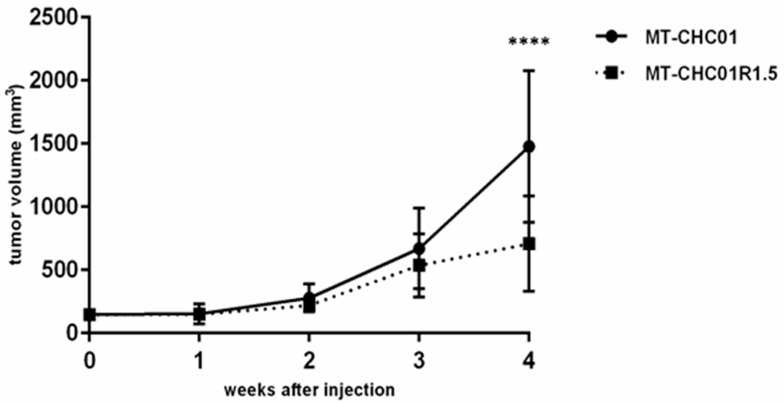
In vivo tumor growth of MT-CHC01 and MT-CHC01R1.5 in NOD/SCID mice. The graph indicates the mean tumor volume (mm^3^) measured weekly (error bars: SD). Six mice for each cell lines were used and three independent experiments were conducted. **** *p* = 0.00001.

Furthermore, in order to investigate the in vivo tumor growth, both MT-CHC01 and MT-CHC01R1.5 cells were subcutaneously injected in mice. As shown in Figure 4, both cell lines were able to give rise to tumors and no statistically significant difference in terms of tumorigenic potential was found in the first three weeks (*p* = 0.4). Indeed, after 3 weeks, tumors reached a volume ranging from 372 to 1242 mm^3^ in MT-CHC01 xenografts and from 282 to 1007 mm^3^ in MT-CHC01R1.5 xenografts. Interestingly, at the fourth week, the growth curves clearly separated in favor of mice inoculated with GEM sensitive cells (*p* = 0.0001; mean volume 1477.9 ± 599.9 and 707.4 ± 376.4 mm^3^ for MT-CHC01 and MT-CHC01R1.5 xenografts, respectively).

### 2.2. Gemcitabine Resistant Cells Acquired Cross-Resistance to Other Chemotherapeutic Agents

**Figure 5:**
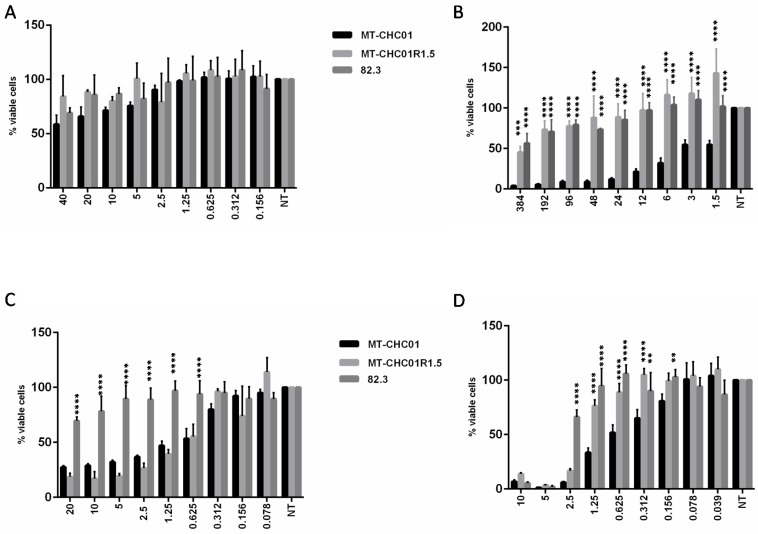
Effects of several chemotherapeutic agents on MT-CHC01, MT-CHC01R1.5, and 82.3 cells. The different drugs ((**A**) Carboplatin; (**B**) 5-FU; (**C**) Oxaliplatin; (**D**) Trabectedin) at the indicated concentrations (for Carboplatin, from 40 to 0.156 μg/mL; for 5-FU, from 384 to 0.75 μM; for Oxaliplatin, from 20 to 0.078 μM; and for Trabectedin, from 10 to 0.039 nM) were added to each cell line. The effect was evaluated by the Cell Titer-Glo assay after 72 h. The values obtained are the mean with SD (bars) of three different experiments. Statistical analysis was performed with Student *t* test. Asterisks indicate a significant *p* value (* *p* < 0.01, ** *p* < 0.001, *** *p* < 0.0001, **** *p* < 0.00001). Statistical analysis was performed comparing resistant cells to MT-CHC01 cells.

Susceptibility to Carboplatin, 5-fluorouracil (5-FU), Oxaliplatin, and Trabectedin on MT-CHC01 and MT-CHC01R1.5 were analyzed by Cell-Titer-Glo assays. As shown in Figure 5A, all the cells were resistant to Carboplatin, while in MT-CHC01R1.5, GEM resistance conferred cross-resistance to 5-FU and Trabectedin agents (Figure 5B,D, respectively). In contrast, no cross-resistance to Oxaliplatin (Figure 5C) was revealed.

The same test on the primary GEM resistant 82.3 cells demonstrated a complete cross-resistance to all evaluated drugs (Figure 5). 

### 2.3. Gemcitabine Resistant Cells Exhibited Specific Expression Profiles with Important Effects on Biological Processes and Molecular Pathways 

To assess the molecular mechanisms underlying the acquisition of GEM resistance, a detailed analysis of the transcriptome in MT-CHC01R1.5 cells versus their corresponding parental counterpart was performed. Among 736 differentially expressed genes, obtained by stringent thresholds, MT-CHC01R1.5 presented 354 down-regulated genes and 382 up-regulated ones (Table S1 and Figure S2). The gene ontology was investigated considering up- and down-regulated genes separately. Among the up-regulated genes, the most significant biological processes were functionally linked to DNA related mechanisms (elongation, replication, and metabolism) and cell cycles (Table S2). This was further confirmed as shown in Table S3 by the analysis of the cellular components and molecular functions. Additionally, process network examination by Metacore software revealed several processes involved in the cell cycle (Core, S phase, G1/S) and DNA damage (DBS, MMR, BER/NER repairs) (Table 1). 

Moreover, a pathway map investigation (Table 2) showed an enrichment of different key regulators of the cell cycle, metabolism of purine and pirymidine, metabolism of folic acid, negative regulation of HIF1-alpha, activation of oxygen reactives upon oxidative stress, cytoskeleton remodeling, immune response, and DNA damage. 

On the contrary, down-regulated genes were globally involved in protein folding, angiogenesis, blood vessel development, xenobiotic metabolism, and, more importantly, in mechanisms related to nutritional changes (i.e., starvation), calcium ion trafficking, hormonal regulation, and hypoxia (Table S4). Table S5 summarizes the significant cellular components and molecular functions enriched for down-regulated genes. Notably, an exploration of the process networks and pathway maps enriched in down-regulated genes showed an augmentation of proteolysis, reproduction, signal transduction mediated by ESR-1 and WNT signaling as well as apoptosis processes, as shown in Table 3 and Table 4, respectively. 

**Figure 6:**
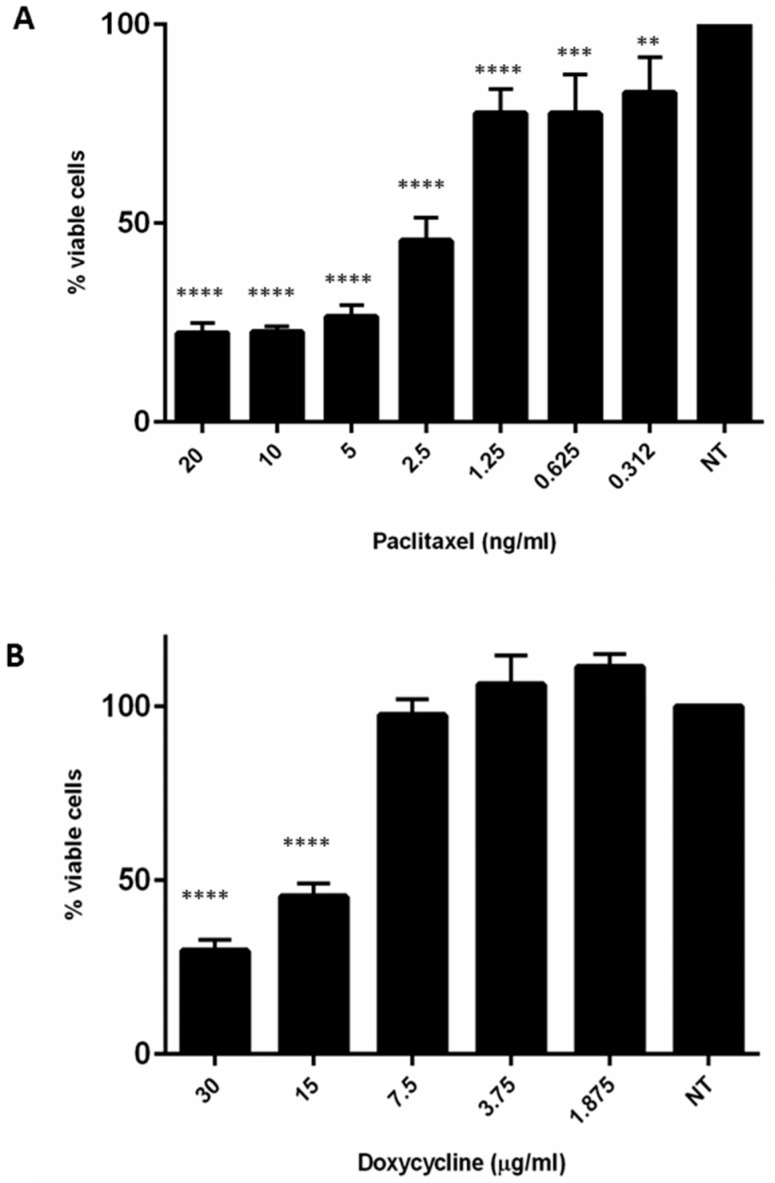
Effects of Paclitaxel (**A**) and Doxycycline (**B**) on MT-CHC01R1.5 cells: MT-CHC01R1.5 cells were sensible at low doses both to Paclitaxel and Doxycycline. The viability was evaluated by Cell Titer Glo assay. All experiments were conducted three times in quadruplicate. ** *p* = 0.001, *** *p* = 0.0001, **** *p* = 0.00001.

Next, a drug prediction analysis based on the first 200 up-regulated genes was performed. Interestingly, eight of these genes (*TUBB2*, *MMP1*, *ADH1*, *TYSY*, *POLA2*, *CyclineE*, *RRM1*, *DDC*) were potentially druggable by chemotherapeutic or antibiotic agents, such as Paclitaxel, Doxycycline, Fomepizole, Pemetrexed, Dacarbazine, Silibinin, Triapine, and Carbidopa, respectively (Table S6). Thereafter, based on this analysis and on literature data [11,12], Paclitaxel and Doxycycline efficacy was tested. Strikingly, both drugs had a statistically significant growth inhibitory effect on MT-CHC01R1.5 cells as shown in Figure 6.

### 2.4. Doxycycline Restored Gemcitabine Sensitivity in Resistant MT-CHC01R1.5 Cells

**Figure 7:**
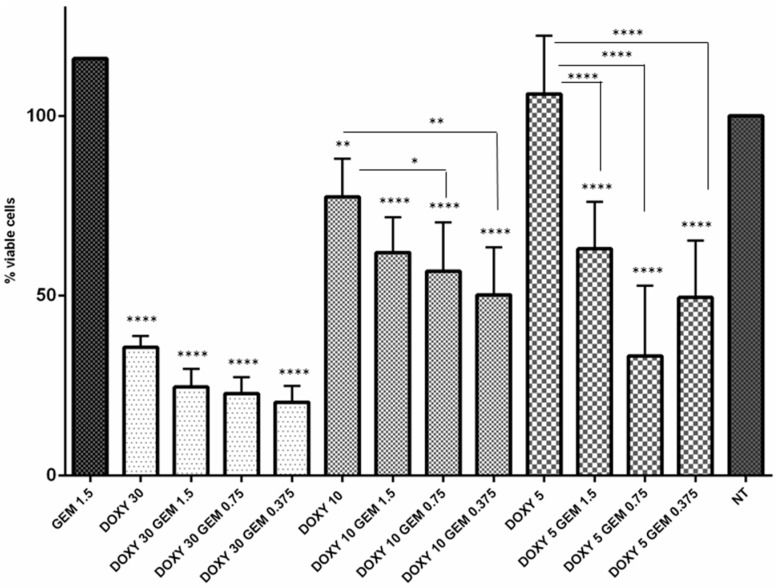
Doxycycline restores the sensitivity of MT-CHC01R1.5 cells to Gemcitabine. Viability of Gemcitabine-resistant MT-CHC01 cells was tested in different culture conditions. GEM 1.5: cells routinely growing in the presence of 1.5 µM Gemcitabine for 72 h; DOXY 30-10-5: cells treated with 30 to 10 or 5 µg/mL of Doxycycline, respectively, for 72 h; DOXY 30-10-5 GEM 1.5-0.75-0.373: cells pre-treated with 30, 10, or 5 µg/mL of Doxycycline for 24 h, followed by 1.5, 0.75, or 0.375 µM Gemcitabine for 72 h; NT: not treated cells. The experiment was repeated three times with four replicates for each dose. Error bars represented means and SD. * *p* = 0.01, ** *p* = 0.001, **** *p* = 0.00001.

As mentioned before, drug prediction studies indicated that MMP1, up-regulated in MT-CHC01R1.5 cells, may be potential druggable by the antibiotic, Doxycycline (DOXY). In order to verify the hypothesis, the sensitivity of MT-CHC01R1.5 cells to GEM in the presence of different DOXY concentrations was assessed. Since that DOXY IC50 value was 17.0 ± 2.2 μg/mL, cells were treated with non-toxic (5–10 μg/mL) and toxic doses (30 μg/mL) (Figure 7). Interestingly, pretreated cells with non-toxic DOXY doses reestablished GEM sensitivity even at very low doses. In contrast, the toxic DOXY dose (30 μg/mL) had a slight effect in restoring GEM susceptibility. Nevertheless, it was able alone to reduce cells’ viability compared to those cells routinely grown in the presence of GEM at a 1.5 μM dose (Figure 7).

### 2.5. Gemcitabine Resistant Cells Displayed a Modulation of Drug Resistance and Epithelial to Mesenchymal Transitions Related Genes

**Figure 8:**
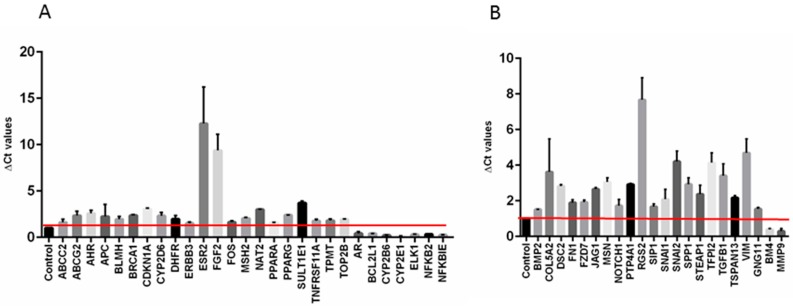
Differentially expressed genes obtained using panels of cancer drug resistance (**A**) and EMT (**B**) related genes. Control: MT-CHC01 parental cells, which represent the reference.

Next, to corroborate our previous in vitro findings, an additional molecular characterization of MT-CHC01R1.5 cells compared to their parental counterpart was achieved by RT2 profiler PCR arrays specific for drug resistance and EMT related pathways. Interestingly, 25% of drug resistance genes in MT-CHC01R1.5 cells were upregulated and these include key molecules, such as *ESR2* (estrogen receptor 2), *FGF2* (fibroblast growth factor 2), and *SULT1E1* (sulfotransferase family 1E member) genes. Only 7 out of 84 (8.3%) were down-regulated (Figure 8A). The same analysis was performed using an EMT related genes panel. Among the 20 up-regulated genes (23.8%) (Figure 8B), the most overexpressed were *COL5A2* (collagen type V alpha 2 chain), *RGS2* (regulator of G-protein signaling 2), *SNAI2* (snail family transcriptional repressor 2), *TFPI2* (TFPI2 tissue factor pathway inhibitor 2), *TGFB1* (transforming growth factor beta 1), and *VIM* (vimentin), thus suggesting an aggressive molecular phenotype of MT-CHC01R1.5 cells. Only 2 out of the 84 genes (2.4%) were down-regulated.

To technically validate the gene expression data, we selected nine genes of the up-regulated panel through microarray and qRT-PCR experiments; some of them overlapped between the two analyses, and the expression of all the selected genes was confirmed. Simultaneously, we evaluated the expression of the same group of genes in a primary cell culture isolated in our laboratory. This culture, named 82.3, was established from a patient primarily resistant to GEM. Interestingly, the same trend in gene expression was found (Table S7).

### 2.6. Methylation Assay

**Figure 9:**
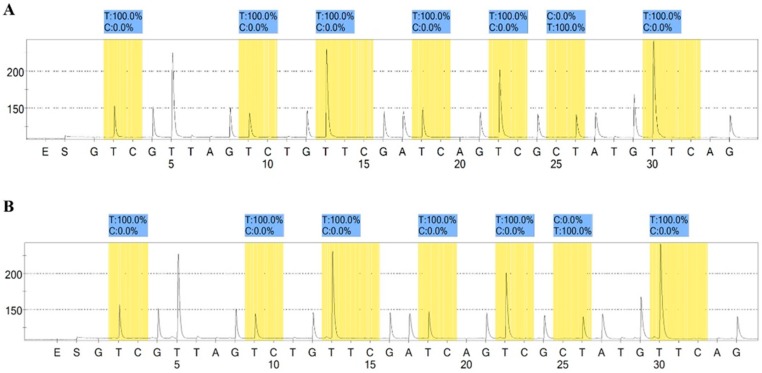
*MGMT* promoter methylation level in (**A**) sensitive and (**B**) resistant cells. The highlighted regions represent the analyzed CpG site of the *MGMT* promoter with the percentage of C showing the level of methylation each site. The experiment was conducted in duplicate.

To evaluate the potential modification in the methylation pattern after the establishment of GEM resistance in the cell line, the well-known *MGMT* promoter methylation marker [13,14] was used to assess the status of both sensitive MT-CHC01 and resistant MT-CHC01R1.5 clones. As shown in Figure 9, neither the sensitive nor the resistant clone showed any methylation in all seven promoter CpG sites (mean percentage of the thymine representing the unmethylated cytosine of 100%), thus indicating that the GEM resistance of MT-CHC01R1.5 cells was not influenced by DNA methylation changes.

## 3. Discussion

In the present study, we investigated the important issue of acquired therapy resistance in human CCA. By continuous long-time exposure to Gemcitabine of sensitive MT-CHC01 cells, final surviving drug-resistant cells, MT-CHC01R1.5 cells, were further functionally and molecularly characterized. Importantly, acquired GEM-resistance was an irreversible phenomenon, as shown in MT-CHC01R1.5 cells when deprived of GEM for 10 days and lately re-exposed at high doses to the drug (up to 10 μM). This is also confirmed by other described CCA multidrug resistant models [15]. In contrast to the “on/off drug exposure system” described in several pancreatic and CCA models [15,16], the irreversible GEM-resistance of MT-CHC01R1.5 cells represents the key feature to ensure a reliable in vitro model for subsequent drug-resistance related analyses.

Biologically, the GEM resistant clone presented an increase in doubling time (25.3 vs. 49.1 h for MT-CHC01 and MT-CHC01R1.5, respectively) as already observed in other GEM-resistant models [15,17,18,19], including CCA. This difference could be due to a cell cycle perturbation, as S phase expansion of MT-CHC01R1.5, which may protect cells from chemotherapeutic activity, but in the meantime, there was a longer phase of DNA repair after GEM damage induction [19]. Concordantly, a statistically significant decrease in the tumor growth of MT-CHC01R1.5 was observed after four weeks from the in vivo injection compared to the parental counterpart.

Despite an early CCA response to GEM/platinum-based combination chemotherapy, most patients relapse as a result of GEM resistance onset that is often associated with a cross-resistance to other chemotherapeutic agents commonly used in the second line of treatment. Indeed, Wattanawongdon and colleagues established two different GEM resistant CCA cell lines, which also showed cross-resistance to 5-FU, Doxorubicin, and Paclitaxel [15]. In our study, we were able to confirm that acquired GEM resistance in MTCHC01R1.5 cells also conferred cross-resistance to other drugs, such as Carboplatin, 5-FU, and Trabectedin, except for Oxaliplatin. Furthermore, we expanded the analysis to primary GEM resistant 82.3 cells that were resistant to all of the already mentioned chemotherapeutic agents, including Oxaliplatin. This development of multidrug resistance suggests that other therapeutic options should be explored.

Recent advances in the treatment of several tumors have implicated the introduction of target therapies, including monoclonal antibodies and tyrosine kinase inhibitors [20]. However, in CCA, these novel therapeutic approaches have failed to provide clinical benefit [4,21,22,23].

The drug prediction analysis applied to the first 200 over-expressed genes in MT-CHC01R1.5 cells highlighted eight genes as potential therapeutic targets of chemotherapy or antibiotics. Based on this evidence, we tested the efficacy of Paclitaxel and Doxycycline, which have as targets TUBB2 and MMP1, respectively. These drugs were able to overcome GEM resistance even at low doses, comparable to those already tested in other cancer models, such as CCA, gallbladder, and ovarian cancers [24,25,26,27]. In particular, pretreatment of cells with non-toxic concentrations of Doxycycline was able to restore sensitivity even at low doses of Gemcitabine on MT-CHC01R1.5 cells.

In a recent study, it was demonstrated that albumin-bound Paclitaxel particles (nab-paclitaxel) exhibited antitumor activity in monotherapy and synergistic activity in combination with GEM treatment in metastatic pancreatic cancer [28]. Nab-paclitaxel was also FDA-approved in advanced breast cancer after failure of previous chemotherapy, and in combination with carboplatin in non-small cell lung cancer [29,30]. In a restricted series of CCA patients that failed first-line treatment, nab-paclitaxel activity was evaluated in combination with GEM or with fluoropyrimidine. The results demonstrated that patients treated with nab-paclitaxel in combination with chemotherapy showed a median overall survival of 9 months and a progression free survival of 6 months; the median time of survival after diagnosis of advanced disease was 21.5 months [31]. Furthermore, in preclinical investigation, Paclitaxel was able to inhibit EMT in CCA cells [24].

Despite the common use of Doxycycline as an antibiotic, there is some evidence of its antitumor activity, in particular, an inhibition of EMT and induction of apoptosis in different cancer types [12,32,33,34]. To date, there are no other data available on the possibility of overcoming GEM resistance with Doxycycline in other tumor models. However, it has been shown in breast cancer models that Doxycycline targets a sub-population of hypoxia-induced cancer stem cells that are Paclitaxel-resistant, overcoming hypoxia-induced drug-resistance [35]. Similarly, Doxycycline was shown to sensitize cells to tumor necrosis factor and Paclitaxel-induced apoptosis in a pancreatic in vitro model [36]. Thus, both Paclitaxel and Doxycycline are promising therapeutic agents in treatment after the first line GEM.

Several scientific studies show that the promotion of drug resistance mechanisms involves alterations in cell membrane transporters and cell distribution within the cell cycle, activation of mechanisms of DNA repair and/or anti-apoptotic processes, as well as modifications in drug metabolism, and EMT. These mechanisms can act independently or in combination through different signal transduction pathways [8].

As a matter of fact, the transcriptomic analysis revealed a perturbation of genes involved in cell cycle, in particular, in phase G1/S (i.e., *MCM*s, histone related genes, *Rad51*, *PCNA*, *POLA2*, *DNA ligase I*, *E2F1/2*), activation of DNA repair mechanisms (histone related genes, *XRCC2*, *Rad51*, *PCNA*, *FEN1*, *RAD54L*, *FANCD2*, *FANCA*, *DNA ligase I*, *ChAF1 subunit A/B*, *RFC5*, *EXO1*, *RFC2*, *POLD cat* (*p125*)), and nucleoside metabolism (*RRM1, RRM2*). Importantly the most significant up-regulated gene was *RRM1*, a well-known biomarker of GEM resistance [37]. Taken together, these data suggest that MT-CHC01R1.5 cells were able to protect themselves from the drug’s cytotoxic effects. Simultaneously, a down-regulation of genes related to apoptosis (SHIP, ActRIIB, c-Fos), regulation of the angiogenetic process (Ephrin-A, Ephrin-A1, IL-15, Angiotensin II/III, HB-EGF, Angiogenin, VEGF-A), and xenobiotic metabolism (CYPs, AKR7A3, GGT1, RORA) was evidenced. The reductions at the mRNA level of the latter two processes were not in line with those described in the literature. Besides the already well-established markers of GEM resistance, such as RRM1, RRM2, and RAD51 [17,38], which were also confirmed in our analysis, we also explored two additional panels of genes (*n* = 168) related to drug resistance and EMT. Among the first panel, the most perturbed genes were *ESR2*, *FGF2*, and *SULT1E1* (up-regulated) and *AR*, *BCL2L1*, *CYP2B6*, *CYP2E1*, *ELK1*, *NFKB2*, *NFKBIE* (down-regulated), while within the EMT gene list, *COL5A2*, *RGS2*, *SNAI2*, *TFPI2*, *TGFB1*, *VIM* (up-regulated), and *BMP4* and *MMP9* (down-regulated) were the best represented in MT-CHC01R1.5 cells. Next, based on a comparison between the qRT-PCR and microarray data, a list of nine up-regulated genes in MT-CHC01R1.5 (*BMP2*, *BRCA1*, *DHFR*, *JAG1*, *MMP1*, *MSH2*, *SPOCK2*, *TYMS*, *VIM*) were selected and further confirmed in another in vitro model established in our group, obtained from an ICC patient resistant to GEM. Notably, some of these genes were already described to be linked to GEM resistance, such as *VIM*, a mesenchymal marker that is overexpressed in pancreatic GEM resistant cell lines [39,40]. *TYMS* played an important role in GEM metabolism; in sensitive cells, its expression was down-regulated by the drug, leading to apoptosis [41]. An up-regulation of this gene could cause an escape of cells from death [42]. High levels of *TYMS* correlated with poor responsiveness to 5-FU [43], and this correlated with MT-CHC01R1.5 unresponsiveness to this drug. *JAG1*, one of the ligands of NOTCH, has a proven role in cancer progression and in particular in EMT [44]. In the work of Wang and colleagues, *JAG1* was overexpressed in a GEM resistant pancreatic cell line compared to its parental counterpart [40]. Moreover, in a recent work, Kim and colleagues demonstrated that in a panel of CCA cell lines, GEM resistance was associated to high levels of simultaneous expression of both APEX1 and Jagged 1 proteins, suggesting these molecules are potential therapeutic targets for CCA chemoresistance [45]. The tumor suppressor *BRCA* gene plays a key role in the repair of fatal DNA double strand breaks and is involved in the development of several tumors [46]. Interestingly, it was demonstrated that the BRCA/FA pathway genes and proteins were upregulated in a GEM resistant model of CCA [47].

Other genes are known to be responsible for the drug resistance phenomenon, but are not directly linked to GEM: For instance, *BMP2*, one of the BMP isoforms, is overexpressed in many cancer types [48,49,50] and its role is controversial. Indeed, it is a tumor suppressor in colon cancer [51], but in ovarian cancer, it promotes in vivo chemotherapy resistance by inhibition of in vitro proliferation [52]. *MMP1* is found to be overexpressed in breast cancer cells resistant to Doxorubicin and is involved in the development of multidrug resistance [53,54]. *MSH2*, a member of the mismatch repair machinery, is found to be up-regulated in Paclitaxel resistance cell lines and, if silenced, its sensitivity is restored [55]. The role of *DHFR* in drug resistance is not well elucidated; Chauhan and colleagues hypothesized that its up-regulation could be implicated in Methotrexate resistance in acute lymphoblastic leukemia [56].

Additionally, our analysis showed an up-regulation of *SPOCK2*, a member of the SPARC family, in resistant cells. This data is in contrast with the work of Sherman-Baust and colleagues describing the down-regulation of mRNA expression in ovarian cancer cell lines resistant to Cisplatin and Doxorubicin [57].

Until now, cell lines remain the most useful and simple tool to study cancer development and drug response/resistance mechanisms.

## 4. Materials and Methods

### 4.1. Drugs

Gemcitabine hydrochloride was obtained from SANDOZ (Novartis Division, Italia), Trabectedin from PharmaMar (Pharma Mar, S.A., Madrid, Spain), 5-FU and Carboplatin from TEVA (Teva Italia srl, Milano, Italy), and Oxaliplatin from SUN Ranbaxy (Sun Pharmaceutical Industries Ltd., Goregaon, Mumbai, India). Paclitaxel and Doxycycline were obtained from Sigma Aldrich (Sigma–Aldrich, St. Louis, MO, USA). All drugs were dissolved in water for injection and aliquoted in different working solutions to avoid degradation.

### 4.2. Establishment of Gemcitabine Resistant Cell Line

The previously characterized CCA cell line, MT-CHC01 [58], was cultured in knockout/DMEM/F-12 complete medium containing 10% fetal bovine serum (FBS) (all from Sigma–Aldrich-Merk, Darmstadt, Germany), 100 U/mL penicillin, and 100 μg/mL streptomycin (Life Technologies Gathersburg, MD, USA). The GEM resistant clone, named MT-CHC01R1.5, was obtained by exposing parental cells intermittently to escalating doses of the drug, starting from 10 nM of GEM to 1.5 μM for 9 months. After another 3 months of being continuously cultured in the presence of 1.5 μM, the MT-CHC01R1.5 cells were considered stable.

### 4.3. Gemcitabine Resistant ICC Primary Cell Culture

The primary cell culture, 82.3, was obtained from a tumor specimen derived from the fourth generation of a patient-derived xenograft (PDX). The PDX was obtained from a tumor sample of a patient who underwent surgical resection for ICC. The biological material was obtained from a patient who has signed the informed consent, following institutional review board-approved protocols (001-IRCC-00IIS-10 “FPO-IRCCS, l’Istituto di Ricovero e Cura a Carattere Scientifico Candiolo (TO), Italy”). Briefly, the tumor was enzymatically digested with collagenase (200 U/mL; Sigma–Aldrich) for 3 h at 37 °C. Single cell suspension was obtained by filtering the supernatant through a 70-μm cell strainer (BD Biosciences, San Jose, CA, USA). Cells were finally re-suspended in a knockout/DMEM/F-12 complete medium in the presence of 20% FBS. After 20 passages, the immunophenotype of 82.3 cells was determined by flow cytometric analysis, evaluating CK19, CK7, and EpCAM. As shown in Figure S3**,** the characteristic epithelial markers are expressed. Moreover, cells are intrinsically resistant to GEM (Figure S4).

### 4.4. Flow Cytometry Analysis

Cells were washed in 1X PBS containing 0.1% bovine serum albumin (BSA, Sigma Aldrich, Saint Louis, MO, USA) and 0.01% sodium-azide. For cell permeabilization, when requested, the Fix and Perm reagent (BD Bioscience Milan Italy) was used following the manufacturer’s instructions. The following antibodies were used: FITC (Fluorescein isothiocyanate) conjugated mouse anti-CK7, CK19 (Abcam Cambridge, UK) and EpCAM (BD). The analysis was performed using Summit software (Beckman Coulter, Indianapolis, IN, USA). For cell cycle analysis, 1 × 10^6^ cells were plated in 10 cm dishes for 24 and 48 h and fixed with 70% ethanol, on ice. After 24 h at −20 °C, ethanol was removed by centrifugation for 5 min at 300× *g* and cells were washed with PBS. The cells were then stained with 1 mL of Propidium Iodide (PI) staining solution (0.1% (*v*/*v*) Triton X-100 (Sigma-Aldrich), 10 μg/mL PI, and 100 μg/mL DNase-free RNase A (Sigma-Aldrich) in phosphate buffer saline (PBS)) and incubated in the dark at 37 °C for 15 min. Flow cytometry was performed using a CyAn™ ADP Flow Cytometer (Beckman Coulter, Indianapolis, IN, USA) and data were analyzed using FlowJo software, Version 7.6.3 (Treestar, Ashland, OR, USA). For each experiment, conducted in triplicate, 15,000 events were counted.

### 4.5. Cell Growth Curve

MT-CHC01 and MT-CHC01R1.5 cells (150,000/well × 2 mL) were seeded in 6-well culture plates (three-wells for each cell line) in the optimal medium. After 24, 48, and 72 h, the cells were detached and viable cells counted using an inverted microscope with 0.2% trypan blue dye. To calculate the population doubling time (DT), we used the following formula: DT = T ln2/ln(Xe/Xb), in which T is the time duration of the culture, Xe is the number of cells at the end of the incubation time, and Xb is the number of cells at the beginning of the incubation time.

### 4.6. Colony Formation Assay

Colonies derived from single cells were obtained by plating an equal number of MT-CHC01 and MT-CHC01R1.5 cells (500/well) in 24-well culture plates in optimal medium and then were incubated at 37 °C for five days. At the end of the period time, the medium was discarded and cells were stained with 0.1% crystal violet (Sigma-Aldrich). The number of colonies was counted for each well. The experiments were conducted three times with three points for each experiment.

### 4.7. Cell Growth Assay

The cell viability test was set up by plating 2500 cells per well into 96-well plates in the appropriate culture medium for 24 h. After 24 h, the cells were treated with escalating doses of GEM (from 10 μM to 0.078 μM). After 72 h, cell viability was evaluated with the Cell Titer-Glo^®^ commercial kit (Promega Italia, Milan, Italy) following the manufacturer’s protocol. The measurement of luminescence was performed through the Glomax microplate reader (Glomax-Multi Detection System, Promega). The IC50 of the drug (concentration of drug needed to inhibit the 50% of growth) was calculated using the Calcusyn program (Biosoft, Cambridge, UK) based on the Chou-Talalay method.

### 4.8. In Vitro Migration and Invasion Assays

To test the motility, the transwell chambers assay was used (1 cm^2^/well, BD Falcon, BD Bioscience, Milan, Italy) either in the absence or in the presence of matrigel (Basement Membrane Matrix; BD Matrigel^TM^ (BD Biosciences, San Jose, CA, USA) to evaluate the migration and invasion ability, respectively. The upper and lower cultures, in serum free conditions, were separated by 8-µm pore size poly-vinyl-pyrrolidone-free polycarbonate filters (BD Falcon). The experiments were carried out in triplicate.

After the incubation period, filters were fixed with methanol and stained with 0.5% crystal violet in 25% methanol; cells on the upper surface of the filters were removed using cotton swabs. Cells invading the lower surface were counted in five random fields and expressed as the number of invading cells per well.

### 4.9. In Vivo Tumor Growth Assay in NOD/SCID Mice

For in vivo studies, NOD (non-obese diabetic)/SCID (severe combined immunodeficient) female mice (4–6 weeks old) of about 20 to 25 gr/each (Charles River Laboratory, Rozzano, Milan, Italy) were maintained under sterile conditions in micro-isolator cages at the animal facilities of the IRCCS-Candiolo. All animal procedures were approved by the Institutional Ethical Committee for Animal Experimentation (Fondazione Piemontese per la Ricerca sul Cancro; 177/2015-PR and 178/2015-PR 24 March 2015) and by the Italian Ministry of Health. In three independent experiments, 6 mice were subcutaneously (s.c.) injected into the right flank under anesthesia (mixture of isoflurane and nitrous oxide) with either 3.0 × 10^6^ of MT-CHC01 or MT-CHC01R1.5 cells in 50% growth factor-reduced BD Matrigel. Tumor diameters were measured weekly after cell injection up to tumor engraftment until 4 weeks after injection.

### 4.10. Gene Expression Analysis (GEP)

Total RNA was extracted from MT-CHC01 parental cells and from the corresponding GEM resistant MT-CHC01R1.5. Briefly, cells at 70% of confluence were lyzed using TRIZOL and scraped in ice. RNA extraction and purification was performed using the Absolutely RNA miRNA kit (Agilent Technologies, Santa Clara, CA 95051, USA), following the manufacturer’s instructions. RNA quantity was evaluated by Nanodrop, while the RNA integrity was evaluated by BioAnalyzer. One hundred ng of total RNA were processed using the Low Input Quick Amp Labeling Kit, one-color kit (Agilent Technologies). Six hundred ng of labeled RNA were hybridized on Whole Human Genome Microarray 4 × 44 K glass arrays. Arrays were scanned and images analyzed by the Feature Extraction Software from Agilent Technologies (version 10.7, Santa Clara, CA 95051, USA); raw data were then analyzed using the LIMMA (LInear Models for Microarray Analysis) package from Bioconductor. A threshold of |LogFC| greater than 2 and adjusted *p*-value < 0.001 were used to select modulated transcripts. Gene ontology analysis was performed using the Database for Annotation, Visualization, and Integrated Discovery (DAVID); processes with a *p*-value < 0.01 being considered as statistically significant. Drug prediction, process network, and pathway map analyses were conducted using the Metacore tool (https://portal.genego.com). Microarray data were deposited in the Gene Expression Omnibus (GSE116118).

### 4.11. RT2 Profiler PCR Array

The Cancer Drug Resistance and the Epithelial to Mesenchymal Transition (EMT) arrays were used (Qiagen, Hilden, Germany); each 96-plate contained 84 genes and controls. Briefly, the RT2 First Strand Kit (Qiagen) was used for the reverse transcription of 0.5 µg of total RNA for each cell line. SYBR Green ROX qPCR Mastermix was used to perform the quantitative real time reaction. Data analysis was conducted using the ∆∆CT methods, as described in the protocol. Genes with |2^−∆∆CT^| values > 0.5 were considered modulated.

### 4.12. qRT-PCR Validation Assay

RNA was reverse-transcribed to cDNA with the high capacity cDNA reverse transcription kit (Applied Biosystem, Waltham, MA 02451, USA). Syber Green Mastermix was used for the round of amplification; in particular, cDNA was used for the detection of *VIM*, *BMP2*, *MMP1*, *JAG1*, *DHFR*, *BRCA1*, *MSH2*, *SPOCK2*, and *TYMS* deregulated genes and the *PGK* housekeeping gene with specific primers. Data analysis was conducted by comparing the gene expression values of MT-CHC01R1.5 with those of the parental cells, after normalization with the housekeeping *PGK*. Primer sequences and product lengths are summarized in Table S8.

### 4.13. MGMT Promoter Methylation Analyses

The methylation status of the *MGMT* promoter was evaluated by bisulfite-PCR and pyrosequencing. DNA bisulfite conversion was conducted using the commercial *MGMT* plus^®^ (Diatech Pharmacogenetics, Jesi, Italy) kit. Briefly, 500 ng of genomic DNA were treated with sodium bisulte, inducing the deamination of the unmethylated cytosine into uracil without modifying the methylated ones. Three hundred ng of converted DNA was than amplified on a RotorGene Q (Qiagen, Hilden, Germany) instrument with a couple of primers encompassing 7 CpG sites of the *MGMT* promoter according to the manufacturer’s instructions; all conversion-derived uracil were replaced by thymine. The PCR condition for the *MGMT* gene was 95 °C for 5 min, 45 cycles of 95 °C for 30 s, 53 °C for 30 s, and 72 °C for 20 s, 72 °C for 5 min, and then green signal acquisition at 60 °C for 20 s. To evaluate the percentage of cytosine (e.g., methylated) or thymine (e.g., unmethylated) in each CpG site, Pyrosequencig analysis was carried out using the PyroMark Q96 ID system (Qiagen). Each sample was loaded two times for pyrosequencing (PSQ) and fully methylated and unmethylated DNA included in the kit were used as positive and negative controls in each experiment. PSQ analysis was performed with PyroMarker CpG software 1.0.11 (Qiagen). The software returns a mean methylation value for each 10 CpG site and the total mean of all 10 CpG sites. The samples were considered methylated when the mean of percentage of the cytosine in the 10 CpG sites was higher than 5%.

### 4.14. Statistical Analysis

The two ways Anova test was used to analyze cell and tumor growth as well as the differences in terms of drug response; a *p*-value less than 0.05 (confidence interval 95%) was considered as statistically significant. The one-way ANOVA was used to compare the response at different doses of chemotherapeutic agents within the same cell line, with a *p*-value less than 0.05 considered as statistically significant (confidence interval 95%). Data are expressed as the mean and standard deviation (SD) (bars) of values from at least triplicate assays. Statistical analysis was performed with Student *t* test. Asterisks indicate a significant *p*-value (* *p* < 0.01, ** *p* < 0.001, *** *p* < 0.0001, **** *p*< 0.00001).

## 5. Conclusions

Here, we provide a molecular and biologically functional characterization of our CCA resistant model by comparison with its parental sensitive counterpart. Further analyses focused on the methylation, microRNA, and proteome profiles as well as tailored functional and in vivo studies will be warranted to explore any possible effect of GEM and the potential targets to overcome this resistance.

## Figures and Tables

**Table 1:** Enrichment by process networks of up-regulated genes.

Networks	*p*-Value	Genes Involved
Cell cycle_S phase	1.2 × 10^−24^	MCM4, Histone H1.5, Histone H4, RFC5, MCM3, CHTF18, Rad51, Cdt1, MCM2, PCNA, FEN1, ORC1L, RFC2, MCM7, ChAF1 subunit A, MCM6, Histone H1, POLD cat (p125), POLA2, DNA ligase I, PRIM1, MCM5, MCM8, E2F1, CDC45L, MCM4/6/7 complex, ChAF1 subunit B
Cell cycle_Core	6.9 × 10^−13^	E2F2, MCM4, MCM3, Cdt1, MCM2, FEN1, Kinase MYT1, ORC1L, MCM7, MCM6, DNA ligase I, MCM5, MCM8, E2F1, CDC45L, MCM4/6/7 complex
DNA damage_DBS repair	2.7 × 10^−7^	Histone H4, XRCC2, Rad51, PCNA, FEN1, ChAF1 subunit A, RAD54L, FANCD2, FANCA, DNA ligase I, ChAF1 subunit B
DNA damage_MMR repair	8.8 × 10^−4^	RFC5, EXO1, PCNA, RFC2, POLD cat (p125)
DNA damage_Checkpoint	9.8 × 10^−4^	RFC5, CIA/ASF1, PCNA, Kinase MYT1, RFC2, FANCD2, E2F1
Cell cycle_G1-S	0.001	Rad51, TYSY, PCNA, b-Myb, UHRF1, Ceb1, FANCD2, E2F1
DNA damage_BER-NER repair	0.002	RFC5, PCNA, FEN1, RFC2, POLD cat (p125), DNA ligase I
Apoptosis_Apoptotic nucleus	0.004	Histone H1.5, Rad51, c-Myb, b-Myb, Histone H1, FANCD2, E2F1
Reproduction_Spermatogenesis, motility and copulation	0.008	Histone H1.1, PKC-beta, Histone H2, Histone H2A, PKC, DDC, Histone H1, FANCA
Cytoskeleton_Regulation of cytoskeleton rearrangement	0.008	Tubulin beta, Vimentin, Talin, PKC, Tubulin beta 1, Tubulin beta 2, Band 4.1-like protein 2

**Table 2:** Enrichment by pathway maps of up-regulated genes.

Maps	*p*-Value	Genes Involved
Cell cycle_Start of DNA replication in early S phase	2.68 × 10^−14^	MCM4, MCM3, Cdt1, MCM2, ORC1L, Histone H1, MCM5, E2F1, CDC45L, MCM4/6/7 complex
Cell cycle_Transition and termination of DNA replication	1.86 × 10^−6^	MCM2, PCNA, FEN1, POLD cat (p125), DNA ligase I
NETosis in SLE	3.7 × 10^−6^	Histone H4, Histone H2, Histone H2A, PKC, Histone H1
dCTP/dUTP metabolism	2.29 × 10^−5^	Ribonucleotide reductase, RRM2, Small RR subunit, RRM1, POLD cat (p125), POLA2
dATP/dITP metabolism	4.8 × 10^−5^	Ribonucleotide reductase, RRM2, Small RR subunit, RRM1, POLD cat (p125), POLA2
Cell cycle_Sister chromatid cohesion	6.3 × 10^−4^	CHTF18, PCNA, Histone H1
Immune response_IFN-alpha/beta signaling via PI3K and NF-kB pathways	6.8 × 10^−4^	DHFR, PCNA, b-Myb, E2F1, ISG15
Cytoskeleton remodeling_Neurofilaments	9.3 × 10^−4^	Tubulin beta, Vimentin, NEFL
Transcription_Effect of Folic acid on genome stability	0.001	DHFR, HCP1, TYSY, ADH1
Immune response_IL-4-induced regulators of cell growth, survival, differentiation and metabolism	0.001	MCM4, MCM6, MMP-13, MCM5
Folic acid metabolism	0.001	C1TC, DHFR, HCP1, SLC19A1
DNA damage_Role of Brca1 and Brca2 in DNA repair	0.001	Rad51, PCNA, FANCD2
Development_Transcription regulation of granulocyte development	0.001	c-Myb, PKC, E2F1
Transcription_Negative regulation of HIF1A function	0.002	MCM3, MCM2, MCM7, MCM5
Inhibition of neutrophil migration by proresolving lipid mediators in COPD	0.002	TLN2, Talin, PKC, cPKC
Signal transduction_HTR2A signaling outside the nervous system	0.002	PKC-beta, PKC, cPKC (conventional), E2F1
DNA damage_Nucleotide excision repair	0.003	PCNA, POLD cat (p125), DNA ligase I
Cell adhesion_Cell-matrix glycoconjugates	0.003	CCL5, MMP-1, ECM1
Development_Gastrin in differentiation of the gastric mucosa	0.003	PKC-beta, PKC, cPKC
Complement pathway disruption in thrombotic microangiopathy	0.003	CCL5, PKC, cPKC
Immune response_IL-1 signaling pathway	0.003	CCL5, SPHK1, MMP-1, MMP-13
Oxidative stress_Activation of NOX1, NOX5, DUOX1 and DUOX2 NADPH Oxidases	0.004	PKC-beta, PKC, cPKC
Development_VEGF signaling and activation	0.004	PKC-beta, SPHK1, PKC
Glomerular injury in Lupus Nephritis	0.005	CCL5, PKC-beta1, MMP-1, PKC-beta2
Apoptosis and survival_DNA-damage-induced apoptosis	0.005	FANCD2, E2F1
Transcription_Assembly of RNA Polymerase II preinitiation complex on TATA-less promoters	0.008	DHFR, PCNA
Immune response_IL-16 signaling pathway	0.009	CCL5, PKC, cPKC
DNA damage_Mismatch repair	0.009	EXO1, PCNA

**Table 3:** Enrichment by process networks of down-regulated genes.

Networks	*p*-Value	Genes Involved
Reproduction_Gonadotropin regulation	0.003	ATF-3, EGR1, HB-EGF, GABA-A receptor epsilon subunit, Adenylate cyclase, FosB, AKR1C3, c-Fos
Proteolysis_Connective tissue degradation	0.004	Matrilysin (MMP-7), Trypsin II, Trypsin, ADAM8, SERPINA3 (ACT), Protein C inhibitor
Reproduction_FSH-beta signaling pathway	0.004	EGR1, TGM2, Adenylate cyclase, VEGF-A, ActRIIB, c-Fos, IBP
Signal transduction_ESR1-membrane pathway	0.005	HB-EGF, Adenylate cyclase, Adenylate cyclase type IV, c-Fos, Caveolin-1
Development_Regulation of angiogenesis	0.007	Ephrin-A, Ephrin-A1, IL-15, Angiotensin II, HB-EGF, Angiogenin, VEGF-A, Angiotensin III
Signal transduction_WNT signaling	0.007	Matrilysin (MMP-7), HES1, WISP2, HB-EGF, Adenylate cyclase, VEGF-A, c-Fos

**Table 4:** Enrichment by pathway maps of down-regulated genes.

Maps	*p*-Value	Genes Involved
Protein folding and maturation_Angiotensin system maturation	2.223 ×10^−8^	Angiotensin (2–10), Angiotensin II, Angiotensin IV, Angiotensin I, Angiotensin (1–7), Angiotensinogen, Angiotensin III, Angiotensin (1–9)
Protein folding and maturation_Posttranslational processing of neuroendocrine peptides	1.017 × 10^−7^	NT, LargeNT, Trypsin, NN, LargeNN, NT/NN
Transcription_HIF-1 targets	4.662 × 10^−5^	TGM2, MCT4, IBP1, Stanniocalcin 2, VEGF-A, Cyclin G2, REDD1
Signal transduction_HTR2A signaling outside the nervous system	9.750 × 10^−5^	EGR1, HB-EGF, Adenylate cyclase, PLD1, HB-EGF (mature), Caveolin-1
Signal transduction_mTORC1 downstream signaling	3.624 × 10^−4^	eIF4A, VEGF-A, PDCD4, eIF4B, ULK1
Immune response_CD16 signaling in NK cells	6.423 × 10^−4^	PLA2, PLD1, SHIP, c-Fos, VAV-1
Reproduction_Gonadotropin-releasing hormone (GnRH) signaling	7.808 × 10^−4^	ATF-3, EGR1, Adenylate cyclase, FosB, c-Fos
L-Tryptophan metabolism (part 1)	0.001	SLC43A1, ASCT2 (SLC1A5), SLC7A8, SLC38A2, SLC6A14
Development_Leptin signaling via JAK/STAT and MAPK cascades	0.001	EGR1, VEGF-A, c-Fos
Apoptosis and survival_Apoptotic Activin A signaling	0.001	SHIP, ActRIIB, c-Fos
Putative pathways of hormone action in neurofibromatosis type 1	0.001	EGR1, EGR2 (Krox20), FosB
Membrane-bound ESR1: interaction with G-proteins signaling	0.002	HB-EGF, Adenylate cyclase, c-Fos, Caveolin-1
Development_Non-genomic action of Retinoic acid in cell differentiation	0.002	TGM2, VEGF-A, c-Fos, VAV-1
IGF family signaling in colorectal cancer	0.003	FosB, VEGF-A, c-Fos, IBP
Regulation of lipid metabolism_FXR-dependent negative-feedback regulation of bile acids concentration	0.003	FGF19, CYP3A4, CYP2B6
Cytoskeleton remodeling_Fibronectin-binding integrins in cell motility	0.004	ITGAV, VAV-1, Caveolin-1
Development_Role of IL-8 in angiogenesis	0.004	HB-EGF, VEGF-A, c-Fos, Caveolin-1
Muscle contraction_Regulation of eNOS activity in endothelial cells	0.004	KLF2, FosB, VEGF-A, Caveolin-1
Development_Angiotensin activation of ERK	0.004	Angiotensin II, HB-EGF, c-Fos
Immune response_IL-6-induced acute-phase response in hepatocytes	0.005	Angiotensinogen, IBP1, c-Fos
Retinol metabolism	0.005	CYP3A5, CYP3A4, CYP2B6, CYP3A7
Immune response_IL-6 signaling pathway via JAK/STAT	0.005	Rac2, VEGF-A, c-Fos, VAV-1
Transport_ACM3 signaling in lacrimal glands	0.006	Mucin 5B, PLD1, Aquaporin 5, Lysozyme
Transcription_P53 signaling pathway	0.007	HSP27, VEGF-A, c-Fos
Role of tumor microenvironment in plexiform neurofibroma formation in neurofibromatosis type 1	0.007	Rac2, VEGF-A, VAV-1
Development_Role of Activin A in cell differentiation and proliferation	0.007	p15, Adenylate cyclase, ActRIIB
Main pathways of Schwann cells transformation in neurofibromatosis type 1	0.008	Rac2, VEGF-A, c-Fos, VAV-1
Development_Angiotensin signaling via PYK2	0.009	Angiotensin II, c-Fos, VAV-1

## Supplementary Materials

The following are available online at https://www.mdpi.com/2072-6694/11/4/519/s1, Figure S1: ATP production of MT-CHC01 and MT-CHC01R1.5 cells, Figure S2: Volcano plot obtained with the deregulated genes in MT-CHC01R1.5 compared to MT-CHC01 parental cells. In blue, down-regulated genes, in red up-regulated genes, Figure S3: Immunophenotyping of 82.3 ICC primary cell culture, Figure S4: Percentage of viable cells after 72 h of gemcitabine exposure in 82.3 primary cell culture, Table S1: Differentially expressed genes in MT-CHC01R1.5 vs. MT-CHC01 parental cells, Table S2: Biological processes (level 5) enriched for up-regulated genes in MTCHC01R1.5 vs. MTCHC01 cells, Table S3: Cellular component and molecular functions enriched by up-regulated genes in MTCHC01R1.5 vs. MTCHC01 cells, Table S4: Pathways enriched for up-regulated genes in MTCHC01R1.5 vs. MTCHC01 cells, Table S5: Pathways enriched for down-regulated genes in MTCHC01R1.5 vs. MTCHC01 cells, Table S6: Drug prediction analysis: Eight potentially druggable genes from chemotherapeutic or antibiotic agents, Table S7: Expression data validation by qRT-PCR in MT-CHC01R1.5 and in 82.3 primary cell culture, Table S8: Primer sequences used for the qRT-PCR experiment of a selected panel of genes.
