# Supplementary material for: Establishment and Characterization of a New Intrahepatic Cholangiocarcinoma Cell Line Resistant to Gemcitabine
Source: Cancers (Basel). 2019 Apr 11;11(4):519. doi: 10.3390/cancers11040519 (PMC6520787; doi:10.3390/cancers11040519)
Supplement: Supplementary file 1 [file cancers-11-00519-s001.zip › cancers-469693-suppl-layout/cancers-469693-suppl.-layout.docx]

Supplementary Material: Establishment and characterization of a new intrahepatic cholangiocarcinoma cell line resistant to gemcitabine

Chiara Varamo, Caterina Peraldo-Neia, Paola Ostano, Marco Basiricò, Chiara Raggi, Paola Bernabei, Tiziana Venesio, Enrico Berrino, Massimo Aglietta, Francesco Leone, Giuliana Cavalloni


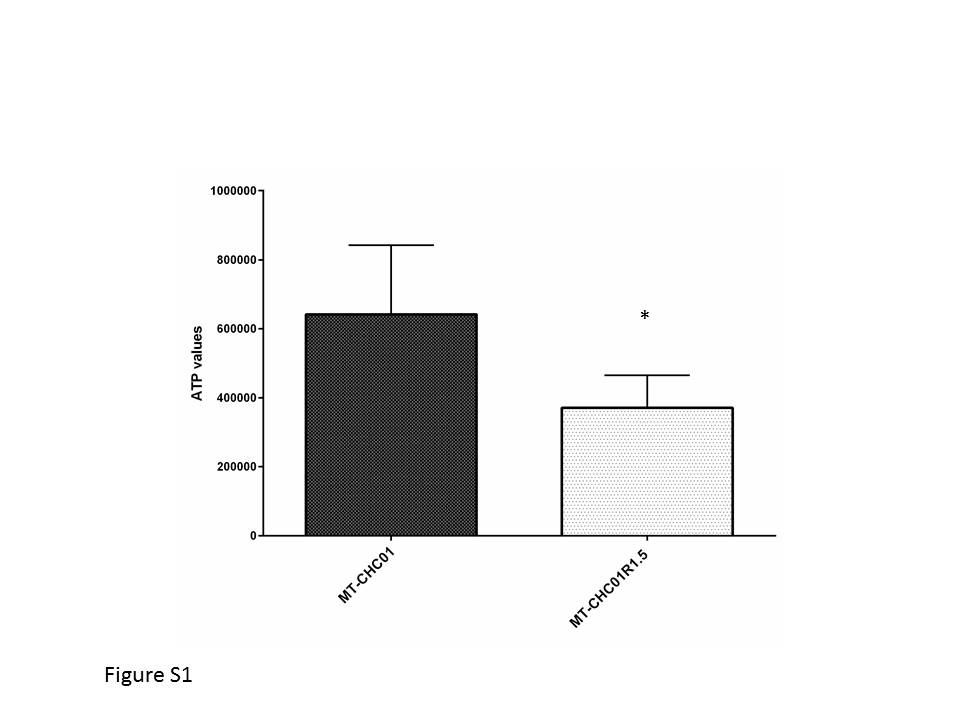


**Figure S1.** ATP production of MT-CHC01 and MT-CHC01R1.5 cells. 2500 cells/well were seeded in optimal culture medium and the ATP quantification was evaluated after 72 h. The experiment was conducted three times in quadruplicate. Error bars represent means and SD. * *p* = 0.01.


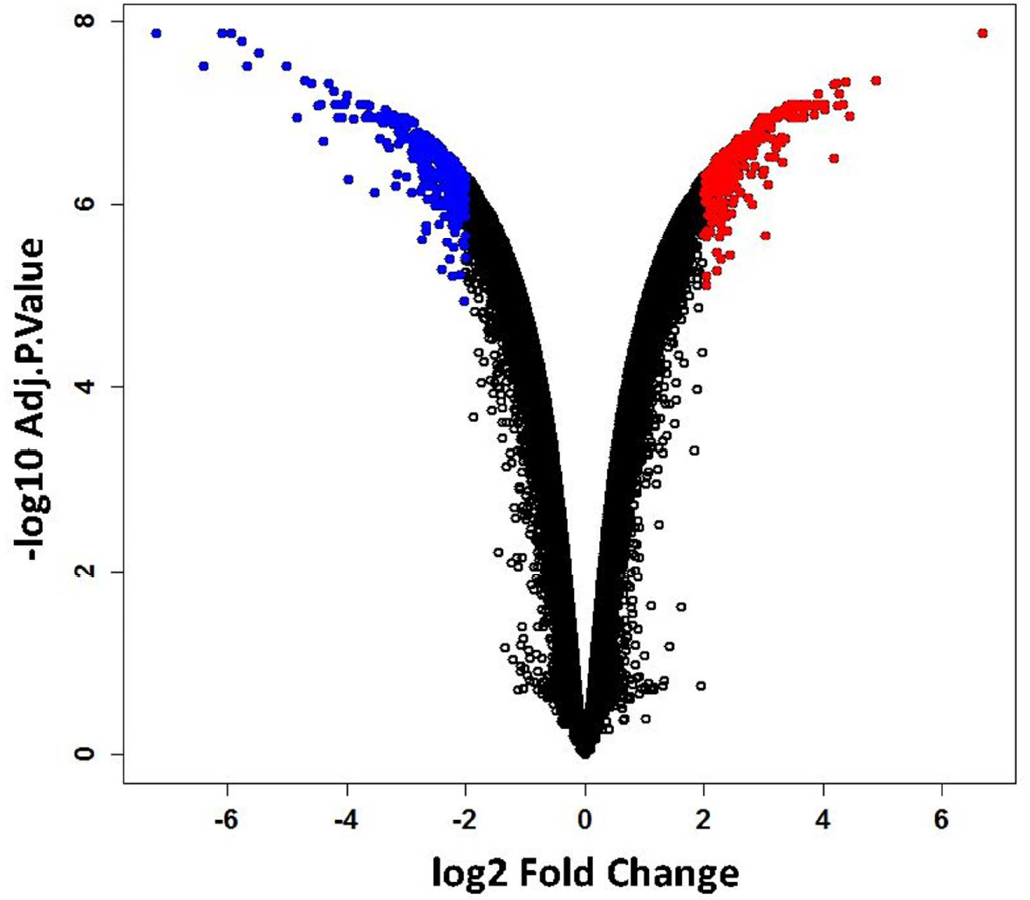


**Figure S2.** Volcano plot obtained with the deregulated genes in MT-CHC01R1.5 compared to MT-CHC01 parental cells. In blue, down-regulated genes, in red up-regulated genes.


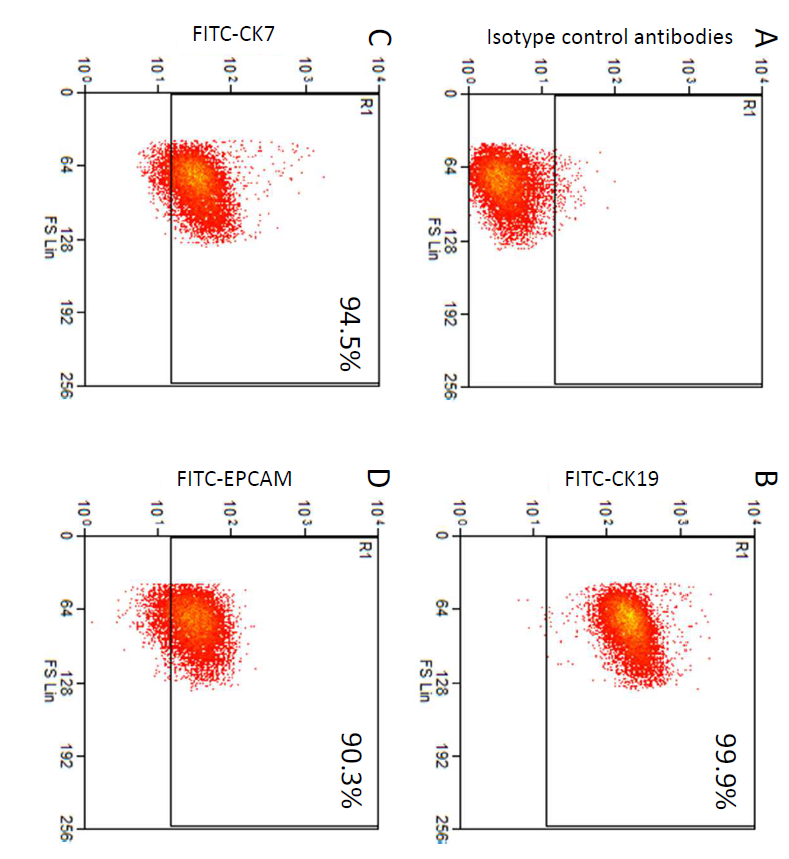


**Figure S3:** Immunophenotyping of 82.3 ICC primary cell culture. Epithelial cell markers are expressed in 82.3 cells. (**A**) Isotype control antibodies: cells were incubated with antibody isotype as primary antibody. Panels B-C-D are representative images of FACS analysis of CK19 (99.9%) (**B**), CK7 (94.5%) (**C**), and EpCAM (90.3%) (**D**).


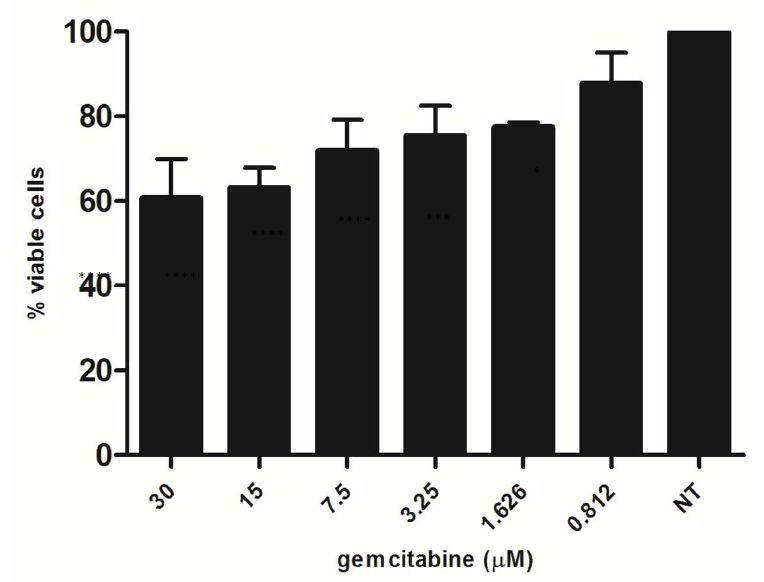


**Figure S4.** Percentage of viable cells after 72 hours of gemcitabine exposure in 82.3 primary cell culture. The effect of gemcitabine is moderate also at high doses.

Tables S1–S6 are in the compressed excel file.

**Table S7.** Expression data validation by qRT-PCR in MT-CHC01R1.5 and in 82.3 primary cell culture.

| **Gene** | **MT-CHC01R1.5** | **82.3** |
| --- | --- | --- |
| BMP2 | 1.97 | 2.8 |
| BRCA1 | 2.93 | 4.97 |
| DHFR | 1.86 | 4.93 |
| JAG1 | 2.65 | 2.88 |
| MMP1 | 57.52 | 263.32 |
| MSH2 | 2.22 | 10.68 |
| SPOCK2 | 7.33 | 4.22 |
| TYMS | 3.12 | 2.55 |
| VIM | 6.52 | 460.2 |

Values represent the gene expression fold increase (2^-ΔΔCt^) in GEM resistant cells compared to the reference (MT-CHC01 GEM sensitive cells).

**Table S8.** Primers used for qRT-PCR of selected genes.

| **Gene name** | **Loci** | **RefSeq** | **Primer Forward** | **Primer Reverse** | **Length (bp)** |
| --- | --- | --- | --- | --- | --- |
| **BMP2** | Chr. 20: 6,767,664-6,780,280 | NM_001200 | CCAGAAACGAGTGGGAAAACA | TCTCGGAAAACCTGAAGCTCT | 101 |
| **BRCA1** | Chr.17: 43,044,295-43,170,245 | NM_007300 | CACCAACATGCCCACAGATC | AAGCCATTGTCCTCTGTCCA | 147 |
| **DHFR** | Chr. 5: 80,626,228-80,654,983 | NM_000791 | TCACCCAGGCCATCTTAAACT | TGGACATCAGAGAGAACACCT | 132 |
| **JAG1** | Chr. 20: 10,637,684-10,673,999 | NM_000214 | TCAATCTACATCGCTTGCGAG | TTTCCTTGATCGGGTTCCCA | 104 |
| **MMP1** | Chr. 11: 102,789,920-102,798,160 | NM_001145938 | CTACCCGGAAGTTGAGCTCA | ACCGGACTTCATCTCTGTCG | 101 |
| **MSH2** | Chr. 2: 47,402,969-47,483,228 | NM_000251 | CAGAAAGCCCTGGAACTTGAG | GGGCATTTGTTTCACCTTGG | 150 |
| **SPOCK2** | Chr. 10: 72,059,035-72,088,773 | NM_001244950 | TGACTGCTGGTGTGTGGAC | TTCTCCTCCTCATCCTCCCA | 135 |
| **TYMS** | Chr. 18: 657,604-673,578 | NM_001071 | CTCACGTACATGATTGCGCA | TGGGAAAGGTCTGGGTTCTC | 135 |
| **VIM** | Chr. 10: 17,228,259-17,237,592 | NM_003380 | GAGTCCACTGAGTACCGGAG | TAGTTAGCAGCTTCAACGGC | 131 |

| 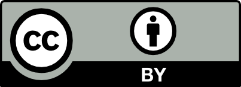 | © 2019 by the authors. Licensee MDPI, Basel, Switzerland. This article is an open access article distributed under the terms and conditions of the Creative Commons Attribution (CC BY) license (http://creativecommons.org/licenses/by/4.0/). |
| --- | --- |
